# Outcomes of Pediatric Osteogenesis Imperfecta Patients Requiring Port‐a‐Cath Placement for Long‐Term Vascular Access

**DOI:** 10.1002/jbm4.10752

**Published:** 2023-04-26

**Authors:** Andrew C White, Jay J Byrd, Makayla Schissel, Elizabeth Strudthoff, Maegen Wallace

**Affiliations:** ^1^ College of Medicine University of Nebraska Medical Center Omaha NE USA; ^2^ Department of Biostatistics, College of Public Health University of Nebraska Medical Center Omaha NE USA; ^3^ The Child Health Research Institute University of Nebraska Medical Center Omaha NE USA; ^4^ Children's Hospital and Medical Center Omaha NE USA; ^5^ Department of Orthopaedic Surgery and Rehabilitation University of Nebraska Medical Center Omaha NE USA

**Keywords:** BISPHOSPHONATES, INFUSIONS, OSTEOGENESIS IMPERFECTA, PORT‐A‐CATH

## Abstract

Intravenous (iv) bisphosphonates are widely used to treat the skeletal manifestations of osteogenesis imperfecta (OI). Obtaining peripheral iv access in pediatric patients with OI is often difficult and traumatic. Although this may be mitigated with surgically placed iv ports (port‐a‐caths), surgeons may be hesitant to perform this procedure on these children because of the lack of safety data. This study aims to gain better insight into the safety and efficacy of port‐a‐cath use in this population and identify risk factors for port‐a‐cath complications. In the present study, we conducted a retrospective cohort analysis of patient characteristics and the incidence of port‐a‐cath‐related complications in children with OI. Fifty‐three port‐a‐caths were placed in 29 children (21 males and 8 females). Of the 29 patients, most are OI type III (*n* = 18), followed by type I (*n* = 4), type IV (*n* = 4), and type V (*n* = 3). At the time of initial port‐a‐cath placement, the median age was 52 months (10–191 months), and the median weight was 7.9 kg (5.1–41.1 kg). Most patients (*n* = 20) weighed less than 10 kg during initial placement. Weight correlated significantly with OI type (*p* = 0.048), sex (*p* = 0.03), and vessel used (*p* = 0.02). Median initial port‐a‐cath longevity was 43 months (1–113 months), and we found no significant difference in port‐a‐cath longevity between sexes, OI types, or vessels used. Most patients (*n* = 19) required multiple port‐a‐cath placements. There is a significant difference (*p* = 0.02) between the number of placements and OI type, with type IV having more than type III. Port‐a‐cath removal was almost always due to mechanical complications (*n* = 19) but also for infection (*n* = 1) and malposition (*n* = 1). Eight patients still had their initial port‐a‐caths in place at the conclusion of this study. These findings indicate that complications associated with port‐a‐cath placement are mild and can be used to safely deliver iv bisphosphonates to pediatric OI patients. © 2023 The Authors. *JBMR Plus* published by Wiley Periodicals LLC on behalf of American Society for Bone and Mineral Research.

## Introduction

Osteogenesis imperfecta (OI) is a genetically, phenotypically, and clinically heterogeneous group of inherited dysplasias with an estimated incidence of 1 per 10,000 individuals.^(^ ^1^ ^)^ The disease predominately manifests in the skeletal system as decreased bone mass, brittle bones, a propensity to fracture, and deformities of the limbs and vertebral column, but it also has a plethora of extraskeletal manifestations.^(^ ^2^ ^)^

Type I collagen is a heterotrimer composed of a triple helix of two α1 chains and one α2 chain and is the most abundant protein in the extracellular matrix of bone and coded for by *COL1A1* and *COL1A2*. Autosomal dominant mutations in these genes were first associated with OI in the late 1970s.^(^ ^3^ ^)^ Decreased quantity of normal type I collagen is associated with less severe OI, whereas the abnormal quality of type I collagen is associated with more severe OI.^(^ ^4^ ^)^

Longtime standard classification of OI types I–IV was proposed by Sillence and colleagues in 1979 based on clinical and radiographic features and inheritance patterns.^(^ ^5^ ^)^ Respective characteristics for type I, type II, type III, and type IV are as follows: mild non‐deforming, perinatally lethal, severe progressively deforming, and phenotypically variable. Types II and III are the most severe, followed by type IV and type I. The discovery of new mutations in genes causing OI led to an adaptation of the Sillence classification by some investigators. This is referred to as the genetic classification, which has 18 types.^(^ ^6^ ^)^ Type V also follows autosomal dominant inheritance but is due to heterozygous mutations in *interferon‐induced transmembrane protein‐5* (*IFITM5*) rather than *COL1A1* or *COL1A2*. Mutations in *IFITM5* increase ectopic ossification, lending to the osteoporotic phenotype of type V OI.^(^ ^6^ ^)^ The Online Mendelian Inheritance in Man database has entries on types I–XXII as of June 2022 (OMIM: PS166200).^(^ ^7^ ^)^

Unfortunately, no cure currently exists for OI. Based on the ubiquity of type I collagen and multisystem clinical manifestation, a multidisciplinary approach is necessary for the management of OI patients. Bisphosphonate therapy is the primary pharmacological strategy utilized to treat these skeletal manifestations.^(^ ^8^ ^)^ Initiating iv nitrogen‐containing bisphosphonate therapy in the early years of life has been shown to decrease pain, bone turnover, and fracture rate, while increasing bone density.^(^ ^9^, ^10^ ^)^ Intravenous pamidronate or zoledronic acid dose frequency schedules vary among centers, from infusions being given every 6 weeks to 6 months.^(^ ^11^ ^)^ Further, monitoring metabolic markers of bone health is imperative to optimize care in these patients. Gaining iv access for bisphosphonate administration and obtaining blood samples can require several attempts. This process is often traumatic and may be mitigated with iv port (port‐a‐cath) placement.

Port‐a‐cath placement in this population is not without concerns, making patients, families, and surgeons hesitant toward their use. Although rare, intraoperative complications in the pediatric population often relate to the number of venipuncture attempts required to place the port‐a‐cath successfully. These include pneumothorax, hydrothorax, cardiac tamponade, arterial puncture, and hemothorax.^(^ ^12^, ^13^ ^)^ The list of postoperative complications is vast. Some potential postoperative complications include bloodstream infections, skin breakdown or other wound complications, mechanical complications such as line occlusion or fracture, and catheter displacement.^(^ ^13^, ^14^, ^15^, ^16^, ^17^ ^)^ Historically, low body weight at the time of port‐a‐cath placement has been a concern for many surgeons, specifically attributable to complications regarding wound healing. However, there is sparse data addressing this concern, and the results are conflicting.^(^ ^18^ ^)^ Furthermore, complications may vary based on the external location and the vessel in which the port‐a‐cath is placed.^(^ ^19^, ^20^, ^21^ ^)^

Multiple studies on central venous catheterization and port‐a‐cath placement outcomes in pediatric patients have been published.^(^ ^15^, ^16^, ^19^, ^21^, ^23^, ^24^, ^26^, ^27^, ^28^ ^)^ However, there is a paucity of literature specific to OI patients.^(^ ^29^ ^)^ The purpose of this study is to investigate the outcomes of port‐a‐cath placement in pediatric patients with OI for long‐term iv access at our center. We aim to facilitate evidence‐based decision‐making by surgeons and other medical providers caring for this population.

## Materials and Methods

### Study population

We performed a retrospective cohort review of all pediatric patients <18 years of age with a diagnosis of OI (ICD code Q78.0) who underwent surgical placement of a port‐a‐cath between 2000 and 2021 (CPT codes 36560, 36561, 36589, and 36590). Twenty‐nine patients were identified from the electronic health record treated at Children's Hospital and Medical Center in Omaha, NE, USA. Approval for the study was obtained from the University of Nebraska Medical Center Institutional Review Board (#0928‐21‐EP).

Patient demographics (sex, age in months, race/ethnicity, weight in kilograms at time of port‐a‐cath placement, and OI type) were collected for each patient. Additionally, data were obtained relating to each port‐a‐cath placement, including the use of antibiotics in the operating room, duration (in months) of placement, the total number of port‐a‐caths placed in each patient, the external location of the port‐a‐cath, the blood vessel used for catheter insertion, complications associated with port‐a‐cath access, and reason for port‐a‐cath removal.

### Port placement and management

At our institution, it is recommended that patients with OI receive iv bisphosphonate treatment. Many patients are treated via peripheral iv access; however, port‐a‐cath placement may be recommended after repeated, difficult, and often traumatic access attempts. Pediatric general surgeons place port‐a‐caths at our institution, and the procedure generally takes 30 to 60 minutes. This can be done alone or while the patient is simultaneously undergoing an orthopedic procedure.

Once in place, port‐a‐caths are accessed for treatment every 8 to 16 weeks for bisphosphonate infusions. Patients are also prescribed monthly port‐a‐cath maintenance. Maintenance includes accessing the port‐a‐cath, assessing the port‐a‐cath for blood return, flushing the port‐a‐cath with normal saline, and instilling a heparin lock.

Port‐a‐caths are removed when they no longer function properly or are no longer needed when the patient can safely tolerate peripheral iv access or require less frequent infusions. When port‐a‐caths malfunction but are still required, they are typically removed and replaced in a single trip to the operating room.

### Statistical analysis

Descriptive statistics were used to summarize the demographic and clinical characteristics of the patients. The number of port‐a‐cath placements was calculated for each of the 29 individuals. Wilcoxon rank sum tests were performed to assess differences between all continuous variables and OI type, weight, and sex categories. OI types with less than 4 individuals (type I and type V) were excluded from the analyses where difference in OI type was compared. The Kruskal–Wallis test was used to assess for differences in the median amount of time the initial port‐a‐cath was in place and port‐a‐cath placement location/vessel used. Only the initial port‐a‐cath is considered in this analysis because it is unclear whether number of port‐a‐caths is associated with placement duration.

Patients were also stratified by weight at the time of port‐a‐cath placement with a cut‐off of 10 kg. Fisher exact test was used to assess the difference between weight categories and all categorical variables. All analyses were conducted in SAS version 9.4 (SAS Institute, Cary, NC, USA). A *p* value <0.05 was considered statistically significant.

## Results

### Study population

From 2000 to 2021, 53 port‐a‐caths were placed in 21 boys (72.4%) and 8 girls (27.6%) in a total of 29 OI patients. Most children in this study had OI type III (*n* = 18, 62%) with types I (*n* = 4, 13.8%), IV (*n* = 4, 13.8%), and V (*n* = 3, 10.3%) being less represented. The median age and weight at the initial port‐a‐cath placement were 52 months (10–191 months) and 7.9 kg (5.1–41.1 kg). More than one port‐a‐cath was required in 21 patients, while the other 8 individuals still had their initial port‐a‐cath in place at the conclusion of this study. Population characteristics are displayed in Table 1.

**Table 1:** Population Characteristics

Deliberate	Total (*N* = 29)
Age (months)	
*N*	29
Mean (SD)	59.6 (45.18)
Median	52.0
Range	10.0–191.0
Weight at surgery	
*N*	29
Mean (SD)	9.7 (6.55)
Median	7.9
Range	5.1–41.1
Type of OI, *n* (%)	
I	4 (13.8%)
III	18 (62.1%)
IV	4 (13.8%)
V	3 (10.3%)
Sex, *n* (%)	
Female	8 (27.6%)
Male	21 (72.4%)
Race/ethnicity, *n* (%)	
Black	1 (3.4%)
Hispanic	3 (10.3%)
Multiple races	4 (13.8%)
White	21 (72.4%)
No. of ports,^a^ *n* (%)	
1	10 (34.5%)
2	15 (51.7%)
3	3 (10.3%)
4	1 (3.4%)

^a^ Refers to the total number of ports placed in a patient during the study period.

### Port‐a‐cath data and outcomes

The median port‐a‐cath longevity was 43 months (1–113 months). The internal jugular vein was used most frequently (55.2%), followed by the subclavian vein (31.0%) and the external jugular vein (13.8%). The most common reason for port‐a‐cath removal was a mechanical complication (90.5%), followed equally by port‐a‐cath misposition and central line infection (4.8%). There was no significant difference in the median time (in months) an initial port‐a‐cath was in place among sex (*p* = 0.65), OI type (*p* = 0.23), or the vessel used (*p* = 0.21). However, the number of port‐a‐cath placements per patient significantly differed by OI type, with type IV OI patients having slightly higher median placement counts than type III OI patients (2.5 versus 2, *p* = 0.02). All initial port‐a‐cath placement characteristics and outcomes data are summarized in Table 2. Mechanical dysfunction (*n* = 19, 90.5%) was the most common complication leading to port‐a‐cath removal or revision. Reasons for port‐a‐cath removal are stratified by vessel used, port‐a‐cath location, and type of OI and are displayed in Table 3.

**Table 2:** Initial Port‐a‐Cath Placement Outcomes

Deliberate	Total (*N* = 29)
Initial port longevity (months)	
*N*	21
Mean (SD)	43.0 (24.35)
Median	43.0
Range	1.0–113.0
Port access frequency (weeks)	
*N*	27
Mean (SD)	9.4 (3.32)
Median	8.0
Range	2.0–16.0
Port location, *n* (%)	
Left lateral chest wall	11 (37.9%)
Right lateral chest wall	10 (34.5%)
Right anterior chest wall	8 (27.6%)
Vessel used, *n* (%)	
Subclavian vein	9 (31.0%)
Internal jugular vein	16 (55.2%)
External jugular vein	4 (13.8%)
Antibiotic use, *n* (%)	
None	16 (72.7%)
Prophylactic	6 (27.3%)
Missing	7
Reason for port removal, *n* (%)	
Mechanical complication	19 (90.5%)
Port mispositioned	1 (4.8%)
Central line infection	1 (4.8%)
Initial port still in place	8

**Table 3:** Reason for Port‐a‐Cath Removal by Vessel, Location, and OI Type

Deliberate	Reason for port removal			Total (*n* = 21)
	Mechanical complication (*n* = 19)	Port mispositioned (*n* = 1)	Central line infection (*n* = 1)	
Vessel used, *n* (%)				
Subclavian vein	6 (31.6%)	1 (100.0%)	0 (0.0%)	7 (33.3%)
Internal jugular vein	10 (52.6%)	0 (0.0%)	1 (100.0%)	11 (52.4%)
External jugular vein	3 (15.8%)	0 (0.0%)	0 (0.0%)	3 (14.3%)
Port location, *n* (%)				
Left lateral chest wall	8 (42.1%)	1 (100.0%)	0 (0.0%)	9 (42.9%)
Right lateral chest wall	7 (36.8%)	0 (0.0%)	1 (100.0%)	8 (38.1%)
Right anterior chest wall	4 (21.1%)	0 (0.0%)	0 (0.0%)	4 (19.0%)
Type of OI, *n* (%)				
I	3 (15.8%)	0 (0.0%)	0 (0.0%)	3 (14.3%)
III	11 (57.9%)	1 (100.0%)	1 (100.0%)	13 (61.9%)
IV	4 (21.1%)	0 (0.0%)	0 (0.0%)	4 (19.0%)
V	1 (5.3%)	0 (0.0%)	0 (0.0%)	1 (4.8%)

Upon initial port‐a‐cath placement, 20 patients weighed <10 kg with a median of 7.3 kg (5.1–9.0 kg). Nine patients weighed ≥10 kg with a median of 11.9 kg (10.2–41.1 kg). There was a significant difference in weight at surgery between sex groups (*p* = 0.03). All 9 individuals who weighed ≥10 kg at initial placement were male. There was a borderline significant difference between weight at surgery and OI type (*p* = 0.048). Of the 18 patients with OI type III, 75% weighed <10 kg upon initial port‐a‐cath placement. There was a significant association between weight categories and where the port‐a‐cath was placed (*p* = 0.02). The majority of those who weighed ≥10 kg had their port‐a‐cath placed in the left lateral chest wall, whereas the majority of those weighing <10 kg had their port‐a‐cath placed in the right lateral chest wall. A significant difference was also detected in the vessel used between the two weight categories (*p* = 0.02). Most port‐a‐cath placements in patients weighing ≥10 kg were in the subclavian vein (66.7%), whereas in the majority of port‐a‐caths in those weighing <10 kg, most port‐a‐caths were placed in the internal jugular vein (70%). The reason for port‐a‐cath removal was not associated with weight at the time of placement (*p* = 1.00). Median port‐a‐cath longevity was longer in those weighing <10 kg than those weighing ≥10 kg (45.0 and 31.5 months, respectively), though this was not statistically significant (*p* = 0.24). Outcomes stratified by weight are displayed in Table 4.

**Table 4:** Port‐a‐Cath Outcomes by Weight

Deliberate	Weight			
	Greater than or equal to 10 kg (*n* = 9)	Less than 10 kg (*n* = 20)	Total (*N* = 29)	*p* Value
Type of OI, *n* (%)				**0.0481** ^**b**^
I	3 (33.3%)	1 (5.0%)	4 (13.8%)	
III	3 (33.3%)	15 (75.0%)	18 (62.1%)	
IV	1 (11.1%)	3 (15.0%)	4 (13.8%)	
V	2 (22.2%)	1 (5.0%)	3 (10.3%)	
Sex, *n* (%)				**0.0332** ^**b**^
Female	0 (0.0%)	8 (40.0%)	8 (27.6%)	
Male	9 (100.0%)	12 (60.0%)	21 (72.4%)	
Initial port longevity (months)				0.2426^a^
*N*	6	15	21	
Mean (SD)	30.7 (18.96)	48.0 (25.04)	43.0 (24.35)	
Median	31.5	45.0	43.0	
Range	1.0–59.0	18.0–113.0	1.0–113.0	
Port location, *n* (%)				**0.0173** ^**b**^
Left lateral chest wall	6 (66.7%)	5 (25.0%)	11 (37.9%)	
Right lateral chest wall	0 (0.0%)	10 (50.0%)	10 (34.5%)	
Right anterior chest wall	3 (33.3%)	5 (25.0%)	8 (27.6%)	
Vessel used, *n* (%)				**0.0160** ^**b**^
Subclavian vein	6 (66.7%)	3 (15.0%)	9 (31.0%)	
Internal jugular vein	2 (22.2%)	14 (70.0%)	16 (55.2%)	
External Jugular vein	1 (11.1%)	3 (15.0%)	4 (13.8%)	
Reason for port removal, *n* (%)				1.0000^b^
Mechanical complication	6 (100.0%)	13 (86.7%)	19 (90.5%)	
Port mispositioned	0 (0.0%)	1 (6.7%)	1 (4.8%)	
Central line infection	0 (0.0%)	1 (6.7%)	1 (4.8%)	
Initial port still in place^c^	3	5	8	

^a^ Wilcoxon rank sum *p* value.

^b^ Fisher exact *p* value.

^**c**^ Not included in analysis because initial port is still in place.

Antibiotics were only used prophylactically in 6 patients. All patients who received prophylactic antibiotics weighed <10 kg upon initial port‐a‐cath placement. None who received antibiotics prophylactically developed a central line infection. The one patient who developed a central line infection required removal of their port‐a‐cath, though this was nearly 75 months after port‐a‐cath placement.

## Discussion

Successfully obtaining and maintaining reliable iv access is imperative in the optimal care for pediatric patients with OI. Surgical placement of port‐a‐caths circumvents morbidity of unsuccessful and traumatic peripheral iv access attempts while also simplifying blood draws and delivery of therapeutics. The present study demonstrates safe and efficacious utilization of port‐a‐caths in children and adolescents with OI in various stages of disease progression. In the existing literature, several notable studies are related to port‐a‐cath outcomes in children.^(^ ^15^, ^16^, ^19^, ^21^, ^22^, ^23^, ^24^, ^25^, ^26^, ^27^, ^28^ ^)^ However, at this time, only one other investigation examining port‐a‐cath outcomes in the OI population has been published to our knowledge.^(^ ^29^ ^)^

Many studies consider port‐a‐cath longevity to be an outcome of interest. One multi‐institution study compared outcomes for port‐a‐caths placed in children <10 kg or ≥10 kg. Most of these port‐a‐caths were placed for chemotherapy, as a result of difficulties in iv access and long‐term infusion necessities. Port‐a‐caths placed in patients <10 kg and ≥10 kg had median longevities of 366.5 and 447 days, respectively.^(^ ^16^ ^)^ Another study evaluated the technical success, risk factors, and complications of port‐a‐caths placed for chemotherapy or hematologic disorders. They reported a mean weight of 8.1 kg and a mean catheter life of 321 days in their population.^(^ ^23^ ^)^ Outcomes of pediatric catheterization in liver transplantation patients grouped by pre‐ and post‐transplant (median weights of 7.5 and 9.9 kg, respectively) showed a median port‐a‐cath life span of 16.1 months (489.7 days).^(^ ^15^ ^)^ Each of these examples lie in stark contrast to our study of OI patients, in which the median port longevity was 43 months (1290 days)—possibly indicative of the longer‐term access necessary in the OI patient population. An investigation of feasibility and complications of venous port‐a‐cath placement by interventional radiology in 21 patients needing port‐a‐caths for oncologic or hematologic disorders found a mean implantation time of 299 days. Although they did not record weights, the mean age of their population was 6 years.^(^ ^26^ ^)^ A single‐institution study by Dillon and colleagues evaluated outcomes of a specific implantation device, most of the time for cancer treatment and less commonly for various systemic diseases. This study also did not evaluate weight; however, mean age was 8.4 years with a mean catheter life of 425 days.^(^ ^27^ ^)^

Complication rate, complication type, and risk factors for complications (both intraoperative and postoperative) are other commonly studied outcomes related to port‐a‐caths. Though the procedure of catheterization has risks, technical success rates of 100% have been reported.^(^ ^23^, ^26^ ^)^ As such, postoperative complication seems more worrisome, with up to 70% of port‐a‐caths having at least one long‐term complication.^(^ ^24^, ^27^ ^)^ Risk factors for postoperative port‐a‐cath complications include but are not limited to low weight at the time of the procedure, external port‐a‐cath location, and vein chosen for catheterization; however, conflicting findings related to these parameters have been reported. Studies of groups <10 kg and ≥10 kg found no statistically significant difference in timing or type of complication and catheter life span.^(^ ^15^, ^23^ ^)^ Two studies have suggested that using the lateral inframammary position and left side may be associated with an increased risk of general and infectious complications.^(^ ^21^, ^23^ ^)^ Complication rates of subclavian vein catheterization have been reported to be up to 43.2%.^(^ ^28^ ^)^ A single‐institution randomized clinical control trial of 280 patients comparing central venous access sites reported increased rates of catheter misposition and infection to be associated with the subclavian and internal jugular veins, respectively. This study found no statistically significant difference in rates of mechanical dysfunction between the veins.^(^ ^19^ ^)^

The port‐a‐cath longevity findings of our present study—median 45 months (1368.8 days) and 31.5 months (985.1 days) in <10 kg and ≥10 kg, respectively—drastically contrast that of previously mentioned investigations but are similar to the only other published study examining pediatric OI patients. A study by Devin and colleagues examined 17 port‐a‐caths placed in pediatric patients with a median weight of 5.8 kg and reported median port longevity of 53.5 months.^(^ ^28^ ^)^ The vast difference in port‐a‐cath longevity observed in these populations may be a product of indication for placement and duration of treatment. Many of these studies were evaluations of port‐a‐caths mostly placed for chemotherapy, suggesting the evaluated population may have a compromised immune system and increased risk of port‐related infection.^(^ ^15^, ^16^, ^23^, ^26^, ^28^ ^)^ Furthermore, a shorter duration of therapy and a more definite treatment period are also likely, in contrast to OI patients who require bisphosphonate infusions for many years, often until skeletal maturity.

Although our results of increased mechanical dysfunction (*n* = 19, 90.5%) are comparable to published findings, we observed central line infections relatively less frequently (*n* = 1, 4.8%) than many other pediatric port‐a‐cath studies. The single patient in the present study who developed a central line infection acquired the infection almost 75 months after port‐a‐cath placement, so it is reasonable to assume that the source of this infection was unrelated to the placement of the port‐a‐cath. Again, the only other OI port‐a‐cath cohort in the literature reported similar postoperative complication rates.^(^ ^29^ ^)^ It is reasonable to assume the same factors affecting port‐a‐cath longevity may correlate with this. An analysis of port‐a‐cath complication rate by OI type is not yet represented in the literature. We found OI type III to have the greatest number of complications (*n* = 11, 57.9%), with types I, IV, and V having fewer but similar incidences. This observation may be attributable to the severity of OI type III but may also be attributed to the overrepresentation of patients with OI type III in our sample. Further investigation with a more representative sample size is warranted before concrete conclusions regarding the associations between complications and OI type are formed.

Although this analysis contains the largest cohort to date of OI patients <10 kg (*n* = 20), ≥10 kg (*n* = 9), and overall (*n* = 29), this study is not without limitations. It is a retrospective chart review performed at a single institution with a limited cohort size. We found no evidence of increased risk of placing port‐a‐caths in patients with OI compared with other port‐a‐cath studies in the pediatric population. An investigation with an increased sample size is needed to better identify predictors of adverse outcomes in OI patients. Furthermore, this study covers a substantial period during which the management of OI and port‐a‐caths may have changed. Nevertheless, the results of the current study are consistent with the findings of Devin and colleagues and further demonstrate the safety and efficacy of port‐a‐caths in pediatric patients with OI.

Our findings contribute to investigations of pediatric catheterization and are a meaningful addition to the limited body of knowledge specific to OI. Although further investigation is warranted to better inform surgeons and other medical professionals who care for these patients, our data support the continued safe and efficacious use of port‐a‐caths in this particularly vulnerable population.

## Author Contributions

**Andrew C White:** Conceptualization; formal analysis; investigation; methodology; writing – original draft; writing – review and editing. **Jay J Byrd:** Conceptualization; formal analysis; investigation; methodology; writing – original draft; writing – review and editing. **Makayla Schissel:** Formal analysis; methodology; writing – original draft; writing – review and editing. **Elizabeth Strudthoff:** Methodology; project administration; writing – review and editing. **Maegen Wallace:** Conceptualization; investigation; methodology; supervision; writing – review and editing.

## Disclosures

MW serves on the Osteogenesis Imperfecta Medical Advisory Council, is a board member for the Jansen's Foundation, served on an advisory board for Ultragenyx, and was a consultant for Novosteo. None of the above conflicted with this work.

### Peer Review

The peer review history for this article is available at https://www.webofscience.com/api/gateway/wos/peer-review/10.1002/jbm4.10752.
